# Prostate dose escalation may positively impact survival in patients with clinically node-positive prostate cancer definitively treated by radiotherapy: surveillance study of the Japanese Radiation Oncology Study Group (JROSG)

**DOI:** 10.1093/jrr/rraf005

**Published:** 2025-03-07

**Authors:** Toshiya Maebayashi, Takashi Mizowaki, Hitoshi Ishikawa, Kiyonao Nakamura, Koji Inaba, Hirofumi Asakura, Hiromitsu Iwata, Satoshi Itasaka, Hiroyuki Wada, Masakuni Sakaguchi, Keiichi Jingu, Takeshi Akiba, Natsuo Tomita, Katsumasa Nakamura, Japanese Radiation Oncology Study Group

**Affiliations:** Department of Radiology, Nihon University School of Medicine, 30-1, Ooyaguchi Kami-cho, Itabashi-ku, Tokyo 173-8610, Japan; Department of Radiation Oncology and Image-Applied Therapy, Graduate School of Medicine, Kyoto University, 54 Kawahara-cho, Shogoin, Sakyo-ku, Kyoto 606-8507, Japan; Hospital of the National Institute of Radiological Sciences, National Institutes for Quantum and Radiological Sciences and Technology, 4-9-7 Anagawa, Inage, Chiba 263-8555, Japan; Department of Radiation Oncology and Image-Applied Therapy, Graduate School of Medicine, Kyoto University, 54 Kawahara-cho, Shogoin, Sakyo-ku, Kyoto 606-8507, Japan; Department of Radiation Oncology, National Cancer Center Hospital, 5-1-1 Tsukiji, Chuo-ku, Tokyo 104-0045, Japan; Radiation and Proton Therapy Center, Shizuoka Cancer Center Hospital, 1007 Shimonagakubo, Nagaizumi-cho, Sunto-gun, Shizuoka 411-8777, Japan; Department of Radiation Oncology, Nagoya Proton Therapy Center, Nagoya City University West Medical Center, 1-1-1 Hirate-cho, Kita-ku, Nagoya 462-8508, Japan; Department of Radiation Oncology, Kurashiki Central Hospital, 1-1-1 Miwa, Kurashiki, Okayama 710-8602, Japan; Department of Radiology, The Jikei University School of Medicine, 3-25-8 Nishi-Shimbashi, Minato-ku, Tokyo 105-8471, Japan; Department of Radiology, Nihon University School of Medicine, 30-1, Ooyaguchi Kami-cho, Itabashi-ku, Tokyo 173-8610, Japan; Department of Radiation Oncology, Tohoku University Graduate School of Medicine, 1-1 Seiryo-chou, Aoba-ku, Sendai 980-8574, Japan; Department of Radiation Oncology, Tokai University School of Medicine, 143 Shimokasuya, Isehara, Kanagawa 259-1193, Japan; Department of Radiology, Nagoya City University Graduate School of Medical Sciences, 1 Kawasumi, Mizuho-cho, Mizuho-ku, Nagoya 467-8601, Japan; Department of Radiation Oncology, Hamamatsu University School of Medicine, 1-20-1 Handayama, Chuo-ku, Hamamatsu city, Shizuoka 431-3192, Japan

**Keywords:** clinically pelvic node-positive prostate cancer, pelvic irradiation, overall survival, androgen-deprivation therapy, biologically effective dose

## Abstract

**Objective:** To retrospectively analyze outcomes of patients who received definitive pelvic irradiation for clinically pelvic node-positive (cT1-4N1M0) prostate cancer (PCa).

**Materials and methods:** Clinical records of 148 patients with cT1-4N1M0 PCa treated with definitive pelvic radiotherapy (RT) between 2011 and 2015 were retrospectively collected from 25 institutions by the Japanese Radiation Oncology Study Group. The median age, initial prostate-specific antigen (PSA) level, and biologically effective dose (BED) to the prostate with α/β of 1.5 Gy were 69 (interquartile range [IQR], 65–74.3) years, 41.5 (IQR, 20.3–89) ng/ml, and 177.3 (IQR, 163.3–182) Gy, respectively. All patients underwent neoadjuvant androgen-deprivation therapy (ADT) for a median duration of 10 months. Most patients (141; 95.2%) received concurrent ADT during the irradiation period. The median duration of adjuvant ADT was 16 (IQR, 5–27.8) months. The Phoenix definition was used to assess biochemical failure.

**Results:** The median follow-up period was 53.5 months (IQR, 41–69.3). The 5-year overall survival (OS) probability was 86.8%. The 5-year biochemical failure-free survival and clinical progression-free survival rates were 69.6% and 76.3%, respectively. Multivariate analysis indicated the BED to the prostate to be a significant prognostic factor for OS. Regarding late adverse events, the estimated cumulative incidences of late Grade 2 or higher gastrointestinal and genitourinary toxicities at 5 years were 8.2% and 5.8%, respectively.

**Conclusion:** Long-term ADT combined with definitive pelvic external beam RT for cT1-4N1M0 PCa leaded to favorable outcomes. Future prospective studies should validate the suggested survival benefit of local dose escalation to the prostate in this cohort.

## INTRODUCTION

Prostate cancer (PCa) is the second most frequently diagnosed malignancy and the fifth leading cause of cancer-related death among men worldwide. In 2022, ~1.5 million new cases and 397 000 deaths were reported globally, accounting for 7.3% of all new cancer cases and 4.1% of all cancer deaths in men [1]. Clinically node-positive (cT1-4N1M0) PCa accounts for ~12% of newly diagnosed cases, as determined by population-based studies and clinical staging methods [2].

Current international guidelines recommend a multimodal approach for the management of cT1-4N1M0 PCa, which includes external-beam radiotherapy (EBRT) to the prostate and pelvic lymph nodes combined with long-term androgen deprivation therapy (ADT) [3, 4]. However, most evidence supporting the management of cT1-4N1M0 PCa is derived from retrospective and observational studies, while randomized controlled trials specifically addressing this patient population remain limited [5].

Notably, although cT1-4N1M0 PCa is classified as stage IV, analyses using the SEER database have shown that cancer-specific survival rates are significantly higher for patients with cT1-4N1M0 PCa than for those with distant metastases [6, 7]. Moreover, recent retrospective and observational studies have consistently demonstrated better survival outcomes for this subgroup of patients. These findings suggest that cT1-4N1M0 PCa represents a distinct subgroup within stage IV disease, with a relatively favorable prognosis despite its advanced classification [8–13].

Therefore, to provide critical data for optimizing radiotherapy (RT) strategies for this condition, we conducted a multicenter, retrospective cohort study within the framework of the Japanese Radiation Oncology Study Group (JROSG). We analyzed the outcomes associated with pelvic irradiation combined with ADT, a treatment approach widely adopted in clinical practice.

## MATERIALS AND METHODS

### Patient population

A questionnaire-based survey for patients with cT1-4N1M0 PCa was conducted at institutions participating in JROSG research. Eligible study subjects were patients with cT1-4N1M0 PCa who had been definitively treated with RT during the period between January 2011 and December 2015. Lymph node metastasis was defined as lymph nodes ≥1 cm in the short axis or lymph nodes showing shrinkage due to neoadjuvant ADT.

### Data collection

In this study, data were collected through a questionnaire-based survey, focusing primarily on patient and treatment characteristics relevant to cT1-4N1M0 PCa. Due to the nature of this survey-based approach, detailed information regarding specific EBRT techniques (e.g. intensity-modulated or 3D conformal RT) was not obtained, and the proportions of cases for which these techniques were used could not therefore be assessed.

### Endpoints

The primary endpoint was overall survival (OS). The secondary endpoints, as specified in the study protocol, were biochemical failure-free survival (BFFS) and clinical progression-free survival (CPFS). In addition to these pre-specified endpoints, we also analyzed PCa-specific survival (PCSS) as an exploratory endpoint, which was not originally included in the study protocol. This post-hoc analysis was performed to provide additional insights into the disease-specific outcomes of the study population.

### Ethical considerations

This surveillance study was approved by the Review Board of the University Hospital Medical Information Network (RK-171010-2).

### Assessments

The definition of biochemical failure (BF) was based on two criteria. The first criterion, known as the Phoenix criterion, is that BF is present if the Prostate-Specific Antigen (PSA) level after treatment rises by 2 ng/ml or more above the lowest PSA level achieved, regardless of the presence of absence of hormone therapy after RT [14]. The second criterion was applied to cases in whom hormone therapy had been continued after RT. In these cases, BF was defined as a PSA increase of at least 25% from a previous measurement taken at least 4 weeks earlier, with the PSA level rising to at least 2.0 ng/ml above the lowest PSA level reached after RT [15]. If either of these criteria was met, recurrence was considered to be present.

Clinical failure (CF) was defined based on imaging findings and included local recurrence of PCa, pelvic and extrapelvic lymph node metastasis, and distant metastasis. However, cases in which PSA elevation was the only evidence of recurrence were excluded as they were considered to have not met the definition of clinical recurrence. Acute and late toxicities were graded according to the National Cancer Institute-Common Terminology Criteria (NCI-CTC), Version4.0 [16].

### Biologically effective dose calculation

For this retrospective study, details on RT schedules, including fractionation and total dose, were collected from participating institutions. The biologically effective dose (BED) for the prostate was calculated using an α/β ratio of 1.5 Gy. This α/β ratio was chosen based on the seminal findings of Brenner and Hall [17], the first to report a low α/β ratio for PCa, and whose approach was subsequently supported by Fowler *et al.* [18]. Although the presence of lymph node metastases might suggest more aggressive disease biology, it was reported that even high-risk disease cases maintained a low α/β ratio of 1.5 [19].

### Statistical analysis

The differences in OS curves were assessed for significance using the Log-rank test with a two-tailed test, and a *P*-value threshold of <0.05 was considered to indicate a statistically significant difference. To identify predictive factors for OS, the following variables were analyzed using their median values: initial PSA value, percentage of positive biopsy cores, age, number of metastatic lymph nodes, BED to the prostate, a boost for metastatic lymph node(s), and T-stage. However, the detailed dose-fractionation data needed to calculate BED1.5 for metastatic lymph nodes were only available in ~one-third of the cases. Therefore, BED1.5 for lymph node metastases was not incorporated into our analysis. The Gleason score was analyzed using a threshold of ≤7 versus ≥8. Missing data were imputed using the mean imputation method. Additionally, the cumulative incidence rates of PCSS, BFFS, and CPFS were estimated using Fine and Gray’s competing risks model. In this framework, non-PCa mortality was treated as a competing risk for PCSS, BFFS and CPFS. Multivariate analysis was performed using a multivariate Cox proportional hazards model to identify predictive factors for OS. Due to the limited number of events, a stepwise regression approach was employed to identify significant prognostic factors. The SPSS 21.0 J statistical software package (SSPS Inc., Chicago, IL, USA) was used for all statistical calculations.

## RESULTS

### Patient characteristics

A preliminary survey was conducted to determine whether definitive RT was being administered to patients with clinically diagnosed cT1-4N1M0 PCa. In all, 34 institutions were confirmed to be providing definitive RT for cT1-4N1M0 PCa patients. Based on these findings, a comprehensive survey was conducted to gather detailed clinical information on cT1-4N1M0 PCa cases treated with definitive RT during the period from January 2011 to December 2015. Consequently, detailed clinical information was collected for 177 cases with cT1-4N1M0 PCa treated with definitive RT at 25 institutions (73.5%). Among these patients, 153 received pelvis irradiation, while 24 were given irradiation confined to the prostate. Eight cases with a follow-up period of less than six months were excluded from the analysis, including five pelvic irradiation and three prostate-confined irradiation cases. Given that the majority of patients received pelvis irradiation, the final analysis included 148 patients who underwent definitive pelvis irradiation with a follow-up period of at least six months. The median follow-up period was 53.5 months (IQR, 41–69.3 months). The patient characteristics are summarized in Table 1.

**Table 1:** Patient characteristics

	*n* = 148
Follow-up (months)	
Median (IQR)	53.5
Range	41.0–69.3
Age (years)	
Median (IQR)	69.0
Range	65.0–74.3
T-Stage	
T1b/1c	1/8
T2a/2b/2c	10/5/6
T3a/3b	47/52
T4	19
Percentage of positive biopsy cores	
Median (IQR)	71.4%
Range	50–100%
Initial PSA (ng/ml)	
Median (IQR)	41.5
Range	20.3–89.0
Gleason score	
6	1
7	29
8	47
9	63
10	8
Number of pelvic lymph node metastases	
1/2/3	85/23/19
4/5/6/≥7/unknown	8/4/1/4/5

Follow-up periods, age, positive biopsy cores and initial PSA are presented as median values, with the corresponding ranges given below. Other data are presented as the number of patients.

**Abbreviations:** IQR; interquartile range, PSA; Prostate-Specific Antigen

### Radiotherapy

Most cases were treated with X-ray EBRT alone, while a few were managed by applying high dose-rate brachytherapy or proton beam therapy as a boost irradiation to the prostate after X-ray EBRT. The doses per fraction (fr) of EBRT were mainly 2 Gy. Pelvic irradiation was defined as RT targeting the primary tumor site and encompassing, at a minimum, the regional pelvic lymphatic drainage pathways, typically including the internal iliac, external iliac, obturator, and presacral lymph nodes. The BED to the prostate and lymph node metastases are summarized in Table 2.

**Table 2:** Details of Radiotherapy

Item	Value
Radiotherapy Techniques	
- X-ray EBRT alone	133 (89.9%)
- HDR brachytherapy + EBRT	4 (2.7%)
- EBRT + proton beam boost	11 (7.4%)
Photon Energy Used in EBRT	
- 6 MV	18 (12.2%)
- 10 MV	56 (37.8%)
- 15 MV	68 (45.9%)
- 16 MV	1 (0.6%)
- 18 MV	5 (3.4%)
Radiation Dose Parameters	
Prostate Irradiation	
- Dose per fraction (Gy)	2.0 (2.0–2.0; 1.8–2.5)
- Number of fractions	37 (35–39; 25–42)
- Total dose (Gy)	74 (70–78; 61–80)
- BED 1.5 (Gy) ^※1^	177.3 (163.3–182.0; 134.2–242.7)
Pelvic Irradiation	
- Dose per fraction (Gy)	2.0 (2.0–2.0; 1.6–2.2)
- Number of fractions	25 (25–25; 20–28)
- Total dose (Gy)	50 (50–50; 40–58.7)
- BED 1.5 (Gy)	116.7 (112.4–116.7; 93.3–136.5)
Irradiation of Lymph Node Metastases ^※2^	
- Dose per fraction (Gy)	2.0 (2.0–2.0; 1.8–2.2)
- Number of fractions	30 (25–36; 25–37)
- Total dose (Gy)	62.9 (50–71; 50–74.8)
- BED 1.5 (Gy)	145.2 (116.7–165.7; 116.7–185.45)

Data are presented as median (IQR; range) or number of patients (%).

**Abbreviations:** EBRT, external beam radiotherapy; HDR, high dose-rate; BED, biologically effective dose (calculated with an α/β ratio of 1.5 Gy); IQR, interquartile range.

※1 Proton beam therapy and high-dose-rate brachytherapy were included as boost modalities.

※2 This parameter was not a primary survey measure; data were available for only 33.9% of patients and should be interpreted with caution.

### Systemic treatment

In all patients, ADT was started prior to RT. The median time from the initiation of ADT to that of RT was 10 months. Most of these patients received concomitant ADT during the irradiation period. The median adjuvant ADT duration was 16 months (interquartile range [IQR], 5–27.8 months). Thirty percent of castration-resistant PCa patients with BF or CF received novel hormone agents or anticancer drugs. The pharmacotherapy utilized in all 148 study subjects is comprehensively summarized in Table 3.

**Table 3:** Details of pharmacotherapy

	n = 148
Period from initiation of ADT to irradiation (months)	
Median (IQR)	10
Range	9–14
Types of pre-irradiation ADT	
CAB	136
Anti-androgen	6
GnRH	5
LHRH	1
Concomitant ADT during RT	141
Types of ADT during RT	
CAB	130
Anti-androgen	3
GnRH	2
LHRH	6
None	7 (due to adverse events)
Duration of post-irradiation ADT (months) (Detailed data were available for 65 patients)	
Median (IQR)	16
Range	5.0–27.8
Types of post-irradiation ADT	
CAB	56
Anti-androgen	1
GnRHa	4
LHRHa	4
Treatment after recurrence	
Enzalutamide	11
Abiraterone	6
Chemotherapy after recurrence	
Docetaxel	18
Cabazitaxel acetonate	3

The period from initiation of ADT to irradiation is presented as a median value, in months, with the corresponding range given below.

**Abbreviations:** IQR; interquartile range, ADT; androgen-deprivation therapy, CAB; combined androgen blockade, GnRHa; gonadotropin releasing hormone antagonist, LHRHa; luteinizing hormone-releasing hormone agonist

### Survivals and prognostic factors for oncologic outcomes

**Fig. 1:**
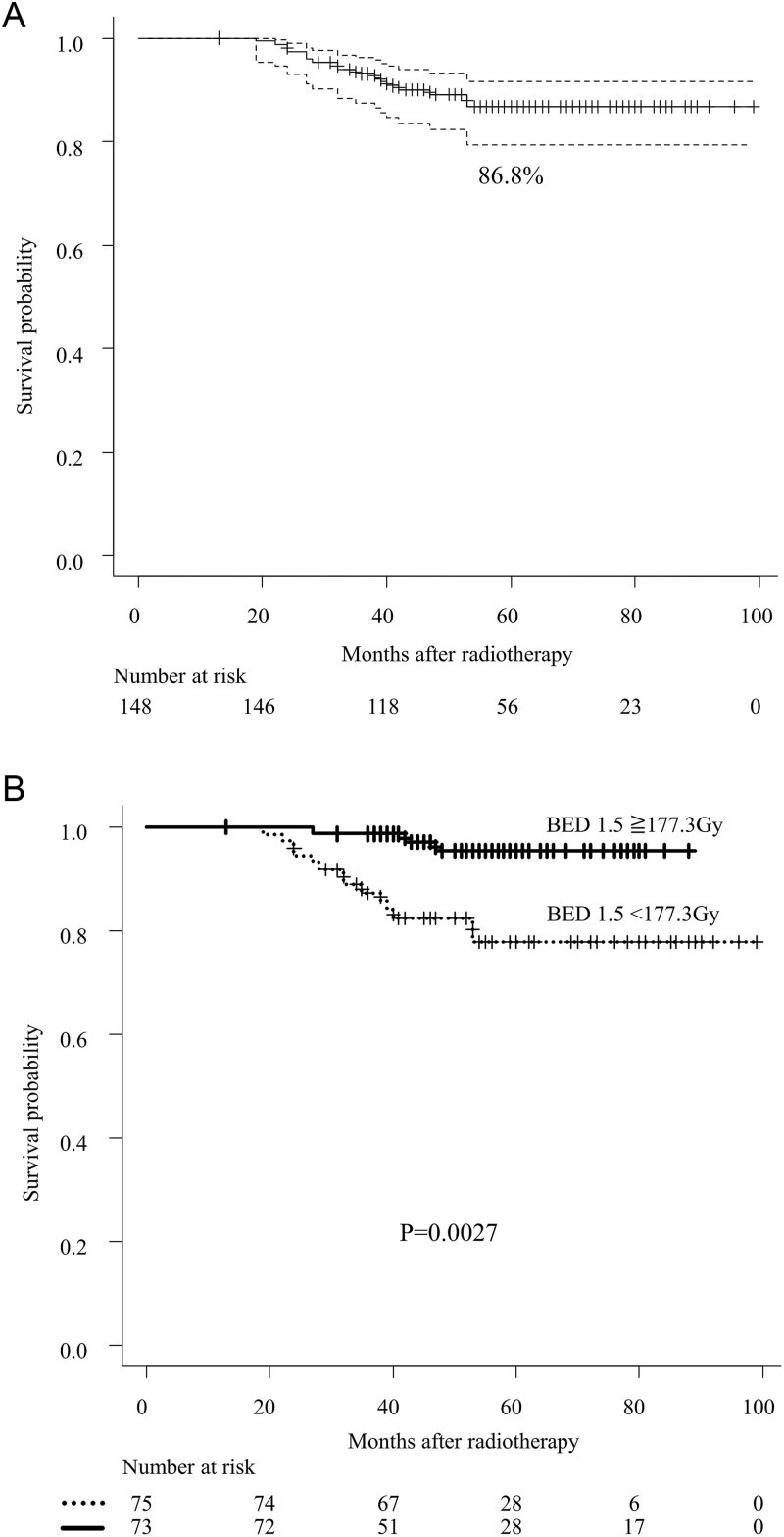
Panel A shows the overall survival of cT1-4N1M0 prostate cancer patients. The black lines denote the pooled reconstructed survival curves, while the dotted lines represent the lower and upper 95% confidence intervals. *P*-values were determined using the stratified log-rank test. **Panel** B illustrates the association between overall survival and the use of BED to the prostate. *P*-values were calculated using the stratified log-rank test. **Abbreviation:** BED; biologically effective dose.

The 5-year OS rate was 86.8% for all patients (Fig. 1A). The 5-year BFFS, CPFS, and PCSS rates were 69.6%, 76.3%, and 90.0%, respectively. BF was observed in 43 patients (29.1%). CF occurred in 28 patients (18.9%), and the median time to the onset of CF from the last day of RT was 28 (IQR, 14–42) months. Cancer-specific death (CSD) occurred in 13 patients (8.8%). The details of BF, CF, and CSD are summarized in Table 4.

**Table 4:** Oncologic and adverse events

	n = 148
Number of biochemical failures	43 (29.1%)
Median (months) (IQR)	30.5
Range (months)	14.5–42.0
Number of clinical failures	28 (18.9%)
bone/ lymph node/ lung	17/ 7/ 3
para-aortic lymph nodes/ liver/ prostate	2/ 2/ 2
Median (months) (IQR)	28
Range (months)	14–42
Adverse events	
Grade2 ≥ acute adverse events	
Gastrointestinal events	18 (10.7%)
Genitourinary events	39 (26.4%)
Leukopenia	7 (4.7%)
Grade2 ≥ late adverse events	
Gastrointestinal events	12 (8.1%)
Median (months) (IQR)	13
Range (months)	11–24
Genitourinary events	9 (6.1%)
Median (months) (IQR)	12
Range (months)	7–40
Cancer-specific death	13 (8.8%)
Alive with disease	25 (16.9%)
Death due to other causes	4 (2.7%)

Numbers of biochemical failures and clinical failures and adverse events are presented as median values, with the corresponding ranges given below. Other data are presented as the number of patients, with percentages indicated in parentheses.

**Abbreviation:** IQR; interquartile range

In the univariate analysis of all patients, both age and BED to the prostate were significantly associated with OS (Fig. 1B and Table 5A). In the multivariable analysis, BED to the prostate remained an independent significant prognostic factor for OS (Table 5A). Given the limited number of events, a stepwise regression approach also identified BED to the prostate as a significant prognostic factor. Conversely, the relationship between boost dose to lymph node metastases and OS did not reach statistical significance in either univariate or multivariate analysis.

**Table 5A:** Multivariable and univariate analyses for overall survival in all patients

	Univariate analysis	Multivariate analysis	
Factor	*P*-value	HR (95% CI)	*P*-value
Initial PSA	0.473	2.147 (0.7061–6.539)	0.178
Percentage of positive biopsy cores	0.662	1.119 (0.545–2.279)	0.756
Age	**0.024**	2.911 (0.981–8.640)	0.054
Gleason Score	0.365	2.373 (0.530–10.650)	0.259
Number of metastatic lymph nodes	0.626	1.116 (0.781–1.595)	0.548
BED to the prostate	**0.003**	5.181 (1.401–19.160)	**0.014**
Boost for metastatic lymph node(s)	0.492	1.098 (0.360–3.347)	0.870
T-stage	0.920	1.540 (0.683–3.475)	0.298
	Stepwise Regression Analysis		
Factor	HR (95% CI)	*P*-value	
BED to the prostate	5.468 (1.57–19.04)	0.008	

**Abbreviations:** PSA; Prostate-Specific Antigen, BED; biologically effective dose, HR; hazard rate, CI; confidence interval

In the univariate competing risk analysis, age and BED to the prostate were significantly associated with PCSS (Table 5B). The Gleason score and number of lymph node metastases were significantly associated with BFFS (Table 5C), while only the number of lymph node metastases was significantly associated with CPFS (Tables 5D). In the multivariable competing risk analysis, age, BED to the prostate, and T stage were independent prognostic factors for PCSS (Table 5B). The Gleason score, number of lymph node metastases, and BED to the prostate were independent prognostic factors for BFFS (Table 5C). For CPFS, PSA, number of lymph node metastases, BED to the prostate, and T stage were independent prognostic factors (Table 5D). Given the limited number of events, a stepwise regression approach confirmed that the significant prognostic factors for PCSS and CPFS identified in the multivariable competing risk analysis were consistent (Table 5B,5D).

**Table 5B:** Multivariable and univariate analyses for prostate cancer specific survival in all patients

	Univariate analysis	Multivariate analysis	
Factor	*P*-value	HR (95% CI)	*P*-value
Initial PSA	0.413	3.587 (0.864–14.89)	0.079
Percentage of positive biopsy cores	0.362	1.268 (0.558–2.879)	0.570
Age	**0.016**	4.345 (1.179–16.010)	**0.027**
Gleason Score	0.247	3.758 (0.488–28.940)	0.200
Number of metastatic lymph nodes	0.234	1.312 (0.930–1.850)	0.120
BED to the prostate	**0.005**	5.832 (1.340–25.380)	**0.019**
Boost for metastatic lymph node(s)	0.476	1.037 (0.299–3.603)	0.950
T-stage	0.580	2.668 (1.030–6.912)	**0.043**
	Stepwise Regression Analysis		
Factor	HR (95% CI)	*P*-value	
BED to the prostate	5.674 (1.292–24.920)	0.022	
Age	3.692 (1.057–12.890)	0.041	

**Abbreviations:** PSA; Prostate-Specific Antigen, BED; biologically effective dose, HR; hazard rate, CI; confidence interval

**Table 5C:** Multivariable and univariate analyses for biochemical failure-free survival in all patients

	Univariate analysis	Multivariate analysis	
Factor	*P*-value	HR (95% CI)	*P*-value
Initial PSA	0.490	2.074 (0.978–4.396)	0.057
Percentage of positive biopsy cores	0.337	1.249 (0.786–1.959)	0.330
Age	0.976	1.409 (0.756–2.625)	0.280
Gleason Score	**0.010**	4.375 (1.311–14.600)	**0.016**
Number of metastatic lymph nodes	**0.001**	1.420 (1.157–1.742)	**0.001**
BED to the prostate	0.123	1.942 (1.026–3.674)	**0.041**
Boost for metastatic lymph node(s)	0.720	1.170 (0.570–2.402)	0.670
T-stage	0.259	1.715 (0.940–3.131)	0.079

**Abbreviations:** PSA; Prostate-Specific Antigen, BED; biologically effective dose, HR; hazard rate, CI; confidence interval

**Table 5D:** Multivariable and univariate analyses for clinical progression-free survival in all patients

	Univariate analysis	Multivariate analysis	
**Factor**	*P*-value	HR (95% CI)	*P*-value
Initial PSA	0.114	3.235 (1.181–8.859)	**0.022**
Percentage of positive biopsy cores	0.378	1.011 (0.590–1.735)	0.970
Age	0.256	1.895 (0.884–4.062)	0.100
Gleason Score	0.062	4.2363(0.908–19.650)	0.066
Number of metastatic lymph nodes	**0.035**	1.341 (1.040–1.727)	**0.023**
BED to the prostate	0.056	2.523 (1.113–8.859)	**0.027**
Boost for metastatic lymph node(s)	0.501	1.286 (0.526–3.145)	0.580
T-stage	0.396	2.047 (1.017–4.119)	0.045
	Stepwise Regression Analysis		
Factor	HR (95% CI)	*P*-value	
Initial PSA	2.972 (1.245–7.091)	0.014	
Number of metastatic lymph nodes	1.308 (1.026–1.669)	0.030	
BED to the prostate	2.353 (1.059–5.231)	0.036	
T-stage	2.001 (1.103–3.603)	0.022	

**Abbreviations:** PSA; Prostate-Specific Antigen, BED; biologically effective dose, HR; hazard rate, CI; confidence interval

As to clinical recurrence, local recurrence within the irradiation field of RT was observed in seven cases (4.7%), and distant metastasis in 22 (14.9%) (Table 6). The nature of pelvic lymph node recurrences—whether progression of pre-existing nodes or new lesions—remains unclear; however, as most cases of recurrent disease was detected more than two years after treatment, the timing suggests them to likely be new lesions.

**Table 6:** Site of Recurrence in 148 patients with cT1-4N1M0 prostate cancer after radiotherapy experiencing clinical recurrence during follow-up

Site (s) of recurrence (%)	Local recurrence (*n* = 2; 1.4%)	Regional lymph nodes recurrence	Distant lymph nodes recurrence	Skeletal recurrence (*n* = 17; 11.5%)	Visceral recurrence and others
		(*n* = 7; 4.7%)	(*n* = 2; 1.4%)		(*n* = 7; 4.7%)
Local region, lung, liver, bone and distant lymph nodes (%)	1 (50.0)	1 (14.3)	1 (50)	1 (5.9)	1 (14.3)
Local region and regional lymph nodes (%)	1 (50.0)	1 (14.3)	-	-	-
Regional lymph nodes only (%)	-	5 (71.4)	-	-	-
Distant lymph nodes and bone (%)	-	-	1 (50)	1 (5.9)	-
Bone only (%)	-	-	-	14 (82.3)	-
Bone, lung and liver (%)	-	-	-	1 (5.9)	1 (14.3)
Lung only (%)	-	-	-	-	2 (28.6)
Other sites (excluding prostate, bones, regional lymph nodes, and lungs) (%)	-	-	-	-	3 (42.9)

### Late adverse events

The estimated 5-year cumulative incidence rates were 8.2% for Grade 2 or higher gastrointestinal (GI) toxicity and 5.8% for genitourinary (GU) toxicity, with Grade 3 events occurring in 2.8% of GI cases and none observed among GU cases. No Grade 4 or higher adverse events were documented. All Grade 2 or higher GI toxicities were attributable to late radiation-induced proctitis resulting in rectal hemorrhage. Among GU toxicities, hematuria was the most frequently observed (six cases), followed by single cases of urinary retention, urinary incontinence, and urinary tract infection.

The median times from the end of RT to the onset of GI and GU toxicities were 13 months (IQR, 11–24) and 12 months (IQR, 7–40), respectively. The details of adverse events are summarized in Table 4.

## DISCUSSION

Regarding the current status of clinical trials for cT1-4N1M0 PCa, the only randomized controlled trial, Radiation Therapy Oncology Group 96–08, was terminated due to insufficient accrual, and there is as yet only one prospective non-randomized comparative study [20]. Additionally, retrospective and observational studies demonstrating detailed long-term outcomes following RT are limited, with only six such studies having been conducted to date [8–13]. Consequently, we conducted a multicenter retrospective study to elucidate details of the RT outcomes and post-treatment status of cT1-4N1M0 PCa patients. Pelvic irradiation was performed in 86.4% of the studied cases, suggesting it to be a common practice. Therefore, we focused on evaluating the outcomes of and prognostic factors for cases in which curative RT included pelvic irradiation. Our study revealed that the 5-year survival rate was relatively favorable at 86.8% when long-term ADT was combined with pelvic irradiation covering the entire lesion. Although high-quality evidence for treating cT1-4N1M0 PCa is lacking, the EAU-ESTRO-SIOG Guidelines [3], the Australian and New Zealand Radiation Oncology Genito-Urinary group recommendations [21], and the NCCN guidelines [4] also endorse treatment with long-term ADT and pelvic irradiation. Recent retrospective studies examining long-term outcomes with similar treatment approaches have also shown favorable five-year survival rates ranging from 86.7% to 92.7% [8, 11, 12]. Therefore, our results, combined with the existing evidence, suggest that aggressive local treatment with curative pelvic irradiation supports the effectiveness of RT for cT1-4N1M0 PCa.

Moreover, factors contributing to improved OS and PCSS included increased radiation dose to the prostate. This finding represents a novel insight into the relationships of the radiation dose with OS and PCSS. This aligns with a meta-analysis on the efficacy of RT to the prostate in metastatic hormone-sensitive PCa, which showed that RT to the prostate combined with long-term ADT improves OS in patients with relatively few distant metastases [22]. Additionally, reports have indicated that dose escalation to the prostate is associated with improved OS in patients with intermediate to high-risk locally advanced PCa [23]. Thus, even in PCa with limited distant metastasis, RT to the prostate is necessary, and dose escalation could improve survival in high-risk groups managed with the aim of achieving curative treatment [24].

Furthermore, the authors of an analysis using the Memorial Sloan Kettering Cancer Center nomogram investigating recurrence and metastasis patterns in N1 PCa patients treated with long-term ADT and pelvic irradiation reported that 24.6% of all cases experienced local recurrence in the prostate, and 74% of patients with biochemical recurrence had local recurrence in the prostate, as detailed by magnetic resonance imaging assessments [9]. Therefore, our study’s finding that higher radiation doses to the prostate are associated with better outcomes suggests that controlling the local tumor within the prostate reduces the risk of distant metastasis and suppresses disease progression.

Additionally, regarding factors associated with BFFS and CPFS, both increased radiation dose to the prostate and the number of lymph node metastases were found to significantly influence recurrence. Previous studies have found PCa cases with two or fewer lymph node metastases to experience favorable biochemical recurrence rates and cancer specific survival [25–27]. Considering these reports, our study results suggest that in cT1-4N1M0 PCa, especially in cases with relatively few lymph node metastases and thereby having the features of oligometastatic disease, aggressive curative treatment may be highly appropriate.

However, despite offering valuable insights, this study has several limitations. First, it was a retrospective multicenter study rather than a prospective trial, which inherently introduces biases and limits the ability to establish causal relationships. Moreover, we did not evaluate variations in RT techniques or specify dose constraints, both of which may have influenced treatment outcomes. Unknown confounding factors might have also affected OS, adding further complexity to the interpretation of our findings. To address missing data, we utilized a mean imputation method; while practical, this approach may have introduced additional uncertainty into the analyses.

Variability in patient backgrounds and treatment approaches across institutions may have further contributed to potential biases. Additionally, the diagnosis of recurrence might have been underestimated, as detailed imaging was not uniformly performed for all patients. For instance, prostate-specific membrane antigen positron emission tomography (PSMA PET) imaging—a highly sensitive modality for detecting recurrence—is not currently covered by the national insurance system in Japan, limiting its routine use. Furthermore, at the time of recurrence with PSA positivity, comprehensive local evaluations of the prostate, such as MRI, were not consistently performed, possibly leading to an underestimation of the true rate of local recurrence. Nonetheless, the comprehensive data collected from major RT centers nationwide enhance the credibility of our findings.

Finally, lymph node metastases are observed in only a subset of patients newly diagnosed with PCa, underscoring the challenges of accurately detecting this advanced stage of the disease [2]. Despite these challenges, traditional diagnostic approaches for lymph node metastases, primarily relying on imaging studies, exhibit limited accuracy, with sensitivities of around 40% and specificities of 80% [28]. The introduction of high-precision diagnostic modalities, such as PSMA PET, is expected to significantly improve the detection of bone metastases, as well as lymph node involvement [29]. This improvement in diagnostic precision would facilitate the identification of true cT1-4N1M0 cases, optimizing the role and outcomes of local treatments like RT. Additionally, intensified systemic therapies, including novel androgen receptor-targeted agents, may be required for some patients. These developments underscore the need for future multicenter prospective trials to refine treatment strategies.

## CONCLUSION

The results obtained in this retrospective cohort study suggest that long-term ADT combined with definitive external beam RT, including pelvic irradiation, leads to favorable outcomes, emphasizing the significance of aggressive local treatment. Moreover, the findings suggest that dose escalation to the prostate may improve the outcomes of patients with cT1-4N1M0 PCa.

## PRESENTATION AT A CONFERENCE

Part of this study was presented at the 62nd Annual Meeting of the American Society for Radiation Oncology (ASTRO), 2022.

## CLINICAL TRIAL INFORMATION

Retrospective cohort study under the auspices of the Japanese Radiation Oncology Study Group (JROSG).
