# Correction to: Prostate dose escalation may positively impact survival in patients with clinically node-positive prostate cancer definitively treated by radiotherapy: surveillance study of the Japanese Radiation Oncology Study Group (JROSG)

**DOI:** 10.1093/jrr/rraf017

**Published:** 2025-05-09

This is a correction to: Toshiya Maebayashi, Takashi Mizowaki, Hitoshi Ishikawa, Kiyonao Nakamura, Koji Inaba, Hirofumi Asakura, Hiromitsu Iwata, Satoshi Itasaka, Hiroyuki Wada, Masakuni Sakaguchi, Keiichi Jingu, Takeshi Akiba, Natsuo Tomita, Katsumasa Nakamura, Japanese Radiation Oncology Study Group, Prostate dose escalation may positively impact survival in patients with clinically node-positive prostate cancer definitively treated by radiotherapy: surveillance study of the Japanese Radiation Oncology Study Group (JROSG), *Journal of Radiation Research*, 2025; https://doi.org/10.1093/jrr/rraf005

In the originally published version of the manuscript marked, emendations to Table 6 had been missed. These are now been emended in the article.

Table 6 should read:

**Table 6:** Site of Recurrence in 148 patients with cT1-4N1M0 prostate cancer after radiotherapy experiencing clinical recurrence during follow-up

Site (s) of recurrence (%)	Local recurrence (*n* = 2; 1.4%)	Regional lymph nodes recurrence	Distant lymph nodes recurrence	Skeletal recurrence (*n* = 17; 11.5%)	Visceral recurrence and others
		(*n* = 7; 4.7%)	(*n* = 2; 1.4%)		(*n* = 7; 4.7%)
Local region, lung, liver, bone and distant lymph nodes (%)	1 (50.0)	1 (14.3)	1 (50)	1 (5.9)	1 (14.3)
Local region and regional lymph nodes (%)	1 (50.0)	1 (14.3)	-	-	-
Regional lymph nodes only (%)	-	5 (71.4)	-	-	-
Distant lymph nodes and bone (%)	-	-	1 (50)	1 (5.9)	-
Bone only (%)	-	-	-	14 (82.3)	-
Bone, lung and liver (%)	-	-	-	1 (5.9)	1 (14.3)
Lung only (%)	-	-	-	-	2 (28.6)
Other sites (excluding prostate, bones, regional lymph nodes, and lungs) (%)	-	-	-	-	3 (42.9)

instead of:

**Table 6:** Site of Recurrence in 148 patients with cT1-4N1M0 prostate cancer after radiotherapy experiencing clinical recurrence during follow-up

Site (s) of recurrence (%)	Local recurrence (*n* = 2; 1.4%)	Regional lymph nodes recurrence (*n* = 7; 4.7%)	Distant lymph nodes recurrence (*n* = 2; 1.4%)	Skeletal recurrence (*n* = 17; 11.5%)	Visceral recurrence and others (*n* = 7; 4.7%)
Local region, lung, liver, bone and distant lymph nodes (%) Local region and regional lymph nodes (%) Regional lymph nodes only (%) Distant lymph nodes and bone (%) Bone only (%) Bone, lung and liver (%) Lung only (%) Other sites (excluding prostate, bones, regional lymph nodes, and lungs) (%)	1 (50.0) 1 (50.0) - - - - - -	1 (14.3) 1 (14.3) 5 (71.4) - - - - -	1 (50) - - 1 (50) - - - -	1 (5.9) - - 1 (5.9) 14 (82.3) 1 (5.9) - -	1 (14.3) - - - - 1 (14.3) 2 (28.6) 3 (42.9)

